# Poly(ADP-ribose) polymerase inhibition suppresses inflammation and promotes recovery from adrenal injury in a rat model of acute necrotizing pancreatitis

**DOI:** 10.1186/s12876-016-0493-5

**Published:** 2016-07-27

**Authors:** Jia Yu, Teng Zuo, Wenhong Deng, Qiao Shi, Peng Ma, Chen Chen, Liang Zhao, Kailiang Zhao, Weixing Wang

**Affiliations:** 1 Department of Hepatobilliary & Laparoscopic Surgery, Renmin Hospital of Wuhan University, Wuhan, 430060 Hubei Province People’s Republic of China; 2 Hubei Key Laboratory for Digestive System Disease, Renmin Hospital of Wuhan University, Wuhan, 430060 Hubei Province People’s Republic of China

**Keywords:** Acute necrotizing pancreatitis, Poly(ADP-ribose) polymerase, 3-Aminobenzamide, Adrenal insufficiency, Adrenal injury, Apoptosis

## Abstract

**Background:**

Poly(ADP-ribose) polymerase (PARP) participates in multi-organ failure in various inflammatory diseases including acute necrotizing pancreatitis (ANP). Since pancreatitis-associated adrenal insufficiency is partly caused by inflammatory damage to the adrenal cortex, we examined whether PARP antagonism could alleviate adrenal insufficiency in a rat model of ANP.

**Methods:**

ANP was induced by retrograde infusion of sodium taurocholate into the bile-pancreatic duct. At 30 min prior to taurocholate infusion, rats were pretreated with the PARP inhibitor 3-Aminobenzamide (3-AB, 20 mg/kg) or vehicle. Pancreatic pathological injury, adrenal histology, neutrophil infiltration, cell apoptosis, and serum corticosterone level were assessed at various times points. Activities of poly(ADP-ribosyl)ated protein (PAR), nuclear factor-kappaB (NF-kB), tumor necrosis factor-α (TNF-α), intercellular adhesion molecule-1 (ICAM-1) and inducible nitric oxide synthase (iNOS) in the adrenal were also examined.

**Results:**

PARP overactivation in ANP rats is associated with reduced serum corticosterone level and marked cellular alterations in adrenocortical tissue. Inflammatory stress caused by ANP reduced adrenal corticosterone release. 3-AB reduced the activation of PARP and inflammatory markers, decreased myeloperoxidase activity, attenuated adrenal morphologic lesions and cells apoptosis, simultaneously improved the impaired adrenal function.

**Conclusions:**

Our data demonstrate the involvement of PARP overactivation in the pathogenesis of adrenal dysfunction after ANP. PARP inhibition may suppress inflammation and promote functional recovery from adrenal injury.

## Background

Relative adrenal insufficiency (RAI), defined as inadequate adrenal corticosteroid production, is common in critically ill patients due to a variety of conditions, including severe acute pancreatitis (SAP) [1–4]. In SAP patients, RAI is associated with increased morbidity and mortality [2]. Dysfunction of the hypothalamic-pituitary-adrenal (HPA) axis is a major contributing factor to adrenal insufficiency under such conditions [5]. Another key factor is organic damage to the adrenal gland. In animal models of acute necrotizing pancreatitis (ANP), adrenal insufficiency could be largely attributed to pathologically verifiable damage to the adrenal gland [6, 7], caused by a variety of pathological processes, including ischemia, hemorrhage, inflammation, and apoptosis [1, 8, 9].

Poly (ADP-ribose) polymerase (PARP) is a key enzyme in DNA repair machinery, and is present in almost all cells [10, 11]. Excessive activation of PARP, however, produces extended chains of ADP-ribose on a variety of nuclear proteins including PARP itself and results in a substantial depletion of intracellular nicotinamide adenine dinucleotide (NAD^+^) and adenosine triphosphate (ATP), leading to cellular dysfunction and death [12]. PARP-1 activity accounts for approximately 90 % of the total poly(ADP-ribose) (PAR) formation [13]. Poly(ADP-ribosyl)ation performs a multitude of biological functions, including regulation of gene expression and gene amplification, cellular differentiation, cellular division and DNA replication, as well as modulating cell apoptosis [11].

PARP participates in inflammatory responses by regulating NF-kB-dependent gene expression, such as TNF-α, Interleukin (IL)-1β, IL-6, ICAM-1 and iNOS [14, 15]. These signaling could affect HPA axis function at the levels of corticotrophin-releasing hormone and adrenocorticotropic hormone activity [16, 17], which in turn affects the immune reciprocally, and eventually the survival of SAP patients. Despite of the pathophysiologic links between stress signals delivered to HPA axis and hormonal regulation in the adrenal gland, the underlying intracellular signaling events causing inadequate adrenal function in ANP are poorly understood.

In the current study, we conducted a comprehensive analysis of adrenal function in a rat model of ANP and examined the potential effects of PARP inhibition in adrenal gland.

## Methods

### Materials

Male Wistar rats (200–250 g) were purchased from the Experimental Animal Center of Hubei Academy of Medical Sciences (Wuhan, China). 3-Aminobenzamid (3-AB), a PARP inhibitor, was obtained from Sigma (#A0788, St. Louis, MO, USA). Other materials, including sodium taurocholate, were obtained from Sigma-Aldrich, except as otherwise noted.

### Experimental design

Adult male rats received a standard diet and water *ad libitum*, and were cared for in adherence with the National Institutes of Health guidelines on the use of laboratory animals. The study was approved by the Ethics Committee of Wuhan University. Rats received retrograde infusion of 5 % TCA (1 mL/kg) into the biliary-pancreatic duct under chloral hydrate (3 ml/kg) anesthesia [18]. 30 min prior to the infusion, rats randomly received either an intravenous bolus of 3-AB (20 mg/kg, dissolved in 5 % DMSO diluted in saline) or vehicle (5 % DMSO). Upon completion of surgical procedures, rats were randomly allocated into the following three groups: (i) TCA + vehicle group (ANP group, *n* = 48); (ii) TCA + 3-AB group (3-AB group, *n* = 48). Rats of ANP and 3-AB groups were sacrificed at 3, 6 (*n* = 8) and 12, 24 h (*n* = 20) after TCA infusion to examine the responses during the acute phase, and at 12 h to examine adrenal injury. A group of 10 rats receiving sham surgery and vehicle treatment was also included as an additional control (CON group).

**Fig. 1:**
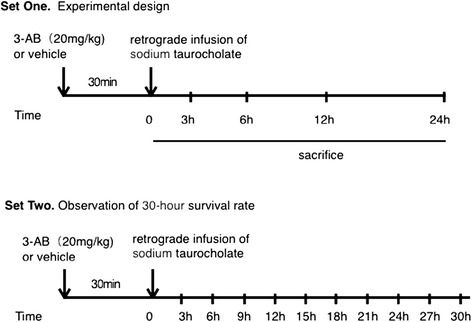
Schematic representation of the experimental protocol. In set one of the experiments, rats received 3-AB or vehicle (i.v.) at 30 min before induction with sodium taurocholate. All rats that survived were sacrificed via intracardiac puncture at respective time points postoperatively. Adrenal and pancreas tissue specimens were collected for ultrastructural morphologic, histopathologic and western analysis. In set two, rats were monitored for a 30-h observation period to examine survival

In an independent set of experiments, rats randomly received treatments described above (*n* = 20 for each of ANP and 3-AB groups), but were monitored for a 30-h observation period to examine survival rate (Fig. 1).

Treatment timing and dosing of 3-AB used were based on the previous study by us to provide beneficial actions against hepatic and renal dysfunction in rats suffering ANP and by others to exert protective effects in experimental models of acute inflammatory response [19, 20].

Surviving rats were sacrificed via intracardiac puncture to obtain pancreatic tissues and adrenal glands. Tissue samples were immersed in 4 % phosphate-buffered formaldehyde for histological examination and terminal deoxynucleotidyl transferase-mediated dUTP-biotin nick end-labeling (TUNEL) assay (*n* = 5). Some adrenal specimen were processed for electron microscopy (*n* = 3), or snap-frozen in liquid nitrogen and stored at −80 °C for subsequent analysis (6 for Western blot, 5 for myeloperoxidase assay).

### Serum assay

The serum was obtained by centrifugation (3000 rpm × 10 mins) of the blood samples. Activities of amylase and lipase were determined by an automatic dry biochemical analyzer (Vitros-350, Johnson & Johnson Co., Calif, USA). Serum corticosterone concentration in rats (cortisol in humans) was measured using an ELISA kit (Cayman Chem. Co., Ann Arbor, MI, USA).

### Histopathologic and TUNEL analysis

Adrenal gland was cut into 2 pieces, one for ultrastructural morphologic analysis using electron microscopy and the other for conventional histopathologic analysis with hematoxylin-eosin staining under a light microscope. Pancreatic histological grading of edema, inflammation, vacuolization and necrosis was quantified by morphometry as previously described [21]. Assessment was conducted by two independent observers.

Adrenocortical hemorrhagic necrosis was examined as described earlier [3]. For each experimental condition, ten magnified fields were analyzed per adrenal section from five different rats. A score from 1 to 4 was given for each adrenocortical profile involving an intersection: grade 1, <25 % necrosis; grade 2, 25–50 % necrosis; grade 3, 50–75 % necrosis; and grade 4, >75 % necrosis. A total score was derived by summation of all 50 scores, with a maximum score of 200, according to the modified method proposed by Chatterjee PK [22].

Cell apoptosis in adrenal cortex was examined using TUNEL staining with an In Situ Cell Death Detection Kit (Roche, Mannheim, Germany). Briefly, 10-μm sections were prepared from paraffin-embedded tissues and deparaffinized. Sections were treated with 20 μg/ml proteinase K for 15 min, blocked in 3 % H_2_O_2_ in methanol for 10 min, permeabilized for 2 min in 0.1 % Triton X-100/sodium citrate at 4 °C, and treated with TUNEL reaction mixture. Sections were exposed to diaminobenzidine for 1 min and dehydrated. Normal nuclei were identified using 1 % methyl green nuclear contrast coloration. Sections were photographed using an Olympus BH-2 microscope. For each section, five fields, chosen at random, were counted.

### Electron microscopy analysis of ultrastructure

Adrenal samples were processed for ultrastructural morphologic study under transmission electron microscope (TEM) as previously described [23]. Specimens were fixed in 2 % glutaraldehyde, post-fixed in 1 % osmium tetroxide, dehydrated in a graded acetone series, and embedded with Epon-812. Ultra-thin sections were stained with uranyl acetate and lead citrate using a standard protocol, and then examined under EM.

### Western blot analysis

PARP activation was examined by analyzing PAR with Western blot as described previously [24]. Nuclear extract of adrenal tissue was prepared by using a Nuclear-Cytosol Extraction Kit (Applygen, Beijing, China) to analyze nuclear NF-kB proteins. Samples were separated on 8 % or 10 % SDS-PAGE, transferred to a nitrocellulose membrane, and probed overnight at 4 °C with one of the following primary antibodies: rabbit monoclonal anti-poly(ADP-ribose) (1:5000, Alexis Biochemicals, Nottingham, UK), NF-kB p65 (1:2000, Cell Signaling, Beverly, USA), goat polyclonal anti-TNF-α, and goat polyclonal anti-ICAM-1 (Santa Cruz, California, USA). Membranes were washed extensively prior to incubation with a HRP-conjugated secondary goat anti-rabbit antibody (1:5000, Pierce, Rockford, IL, USA). Protein bands were visualized using enhanced chemiluminescence, and analyzed with densitometry (Quantity One 4.5.0 software, Bio-Rad Laboratories, Richmond, CA). Sample protein concentration was determined using a BCA method.

### Myeloperoxidase determination

Adrenal MPO activity was examined as described previously [25]. Samples were weighed and homogenized in 1:19 (w/v) in ice-cold homogenization buffer. MPO activity was analyzed with a commercial assay kit (Jiancheng Co., Nanjing, China), and presented as units/g of wet tissue.

### Statistical analysis

All values are expressed as mean ± SD for n observations. In the in vivo studies, *n* represents the number of animal subjects. In the experiments involving histology or immunohistochemistry, the figures shown are representative of at least 3 experiments performed on different days. Statistical analysis was performed using one-way ANOVA, followed by Bonferroni post-hoc test for multiple comparisons. The Kaplan and Meier method was used for analyzing survival rates. Statistically significance was defined as *P* < 0.05.

## Results

### PARP was activated in the adrenal glands after ANP

**Fig. 2:**
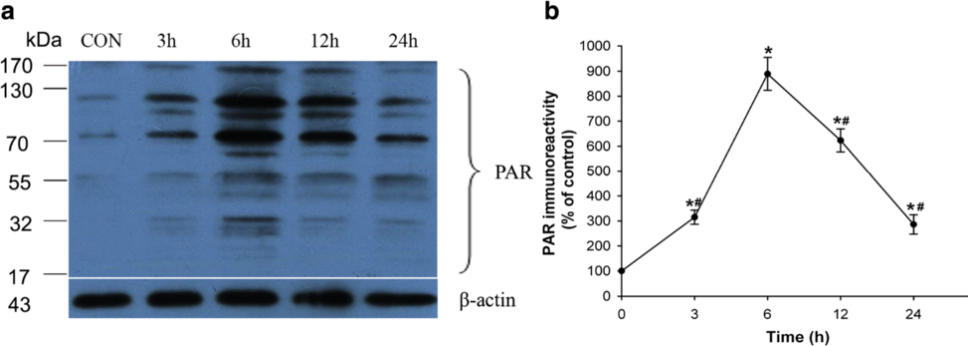
Adrenal poly(ADP-ribosyl)ated proteins in ANP rats. **a** The time course of PAR formation in rat adrenal after pancreatitis. **b** Quantitative representation of PAR at various times after pancreatitis. Values represent mean ± SD (*n* = 3). CON, sham surgery + vehicle; ANP, ANP + vehicle; 3-AB, ANP + 3-AB. *P < 0.05 vs. CON group

Western blot revealed increased Poly(ADP-ribosyl)ated protein, as early as 3 h after TCA retrograde infusion (Fig. 2). The increase peaked at 6 h, and then gradually decreased at 12–24 h to a level higher than control level.

### PARP inhibition reduced pancreatic enzymes and histology

**Fig. 3:**
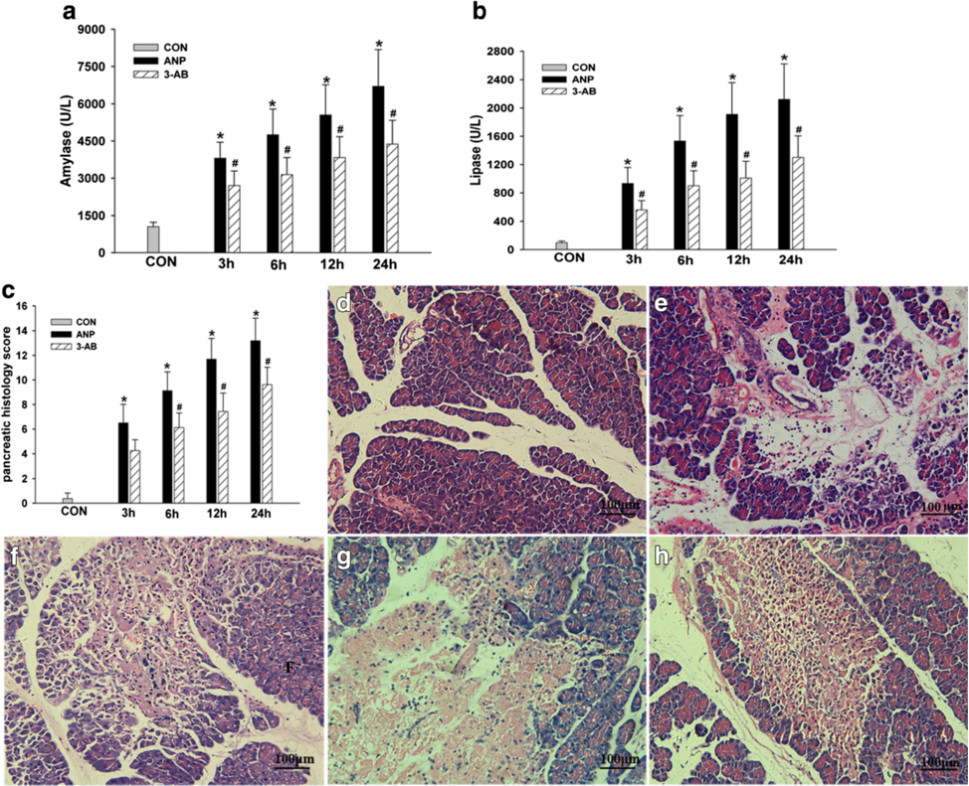
Alterations in serum amylase (**a**) and lipase (**b**) levels. Values represent mean ± SD, *n* = 6–10. *P < 0.05 vs. CON group, ^#^ *P* < 0.05 vs. ANP group. Morphologic changes of rat pancreas in CON (**d**), ANP 3 h (**e**), ANP 6 h (**f**), ANP 12 h (**g**), 3-AB-treated (**h**) rats are shown. No histological alterations were observed in the pancreas tissues from CON group (**d**). Acute damage of the pancreas was observed histologically in ANP rats (**e**-**g**). 3-AB significantly reduced the extent and severity of the histological signs of pancreas (H). Comparison of the pathological score of pancreas (**c**). Figure is representative of at least 3 experiments performed on different experimental days. Bar = 100 μm. Original magnification: ×200

TCA retrograde infusion increased serum amylase and lipase in a time-dependent manner (Fig. 3a-b). 3-AB pretreatment attenuated such increases at all time points of observation. Control rats showed little morphological changes of pancreatic injury except for mild interstitial edema. A substantial increase of pancreatic morphological changes was found in after TCA infusion (Fig. 3e-g). Pancreatic damage was attenuated by 3-AB, as shown histologically in Fig. 3h and by the scores in Fig. 3c.

### PARP inhibition alleviated adrenal insufficiency, histology and neutrophil infiltration

**Fig. 4:**
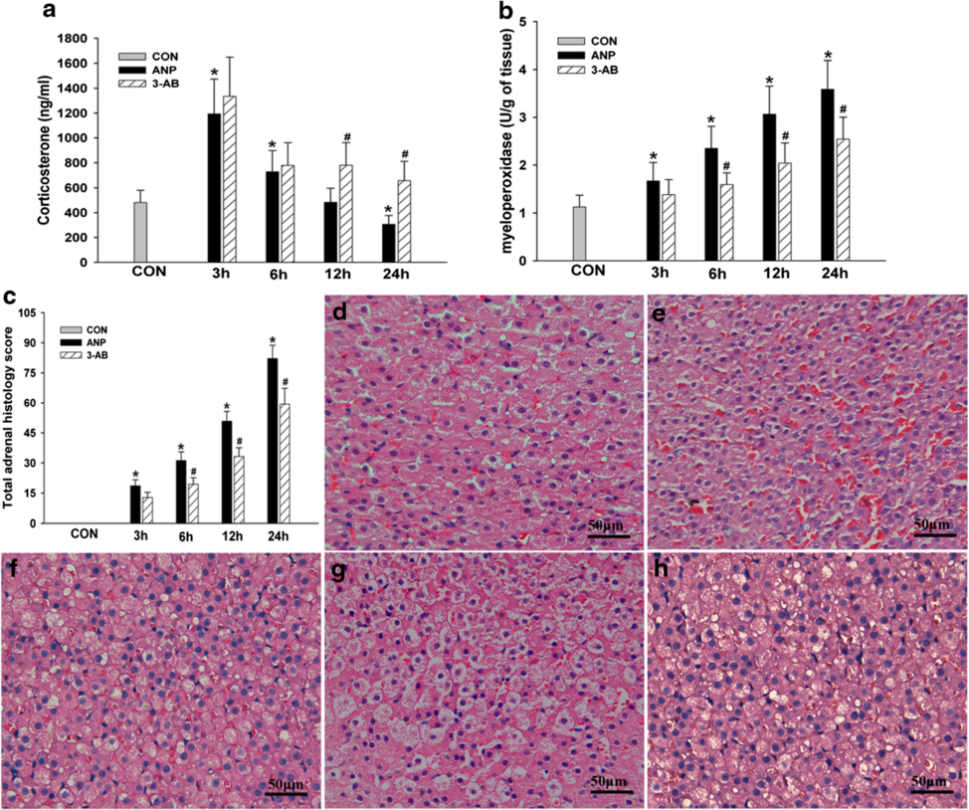
PARP inhibition reduced adrenal insufficiency (**a**) and neutrophil infiltration (**b**) after ANP (*n* = 5). Morphologic changes of rat adrenals in control (**d**), ANP 3 h (**e**), ANP 6 h (**f**), ANP 12 h (**g**), 3-AB-treated 12 h (**h**) rats are shown. No histological alterations were observed in the adrenal tissues from control rats (**d**). 3-AB significantly reduced the severity of the histological signs of adrenal injury (**f**). Comparison of the total histology score of adrenal (**c**). Figure is representative of at least 3 experiments performed on different experimental days. Bar = 50 μm. Original magnification: ×400. *P < 0.05 vs. CON group, ^#^ *P* < 0.05 vs. ANP group

Corticosterone concentration increased immediately after ANP, peaked after 3 h, and decreased to a level significantly lower than the baseline at 24 h. 3-AB pretreatment did not affect corticosterone concentration at 3 and 6 h. However, an increase of corticosterone at 12 and 24 h was apparent in comparison to rats receiving TCA retrograde infusion and vehicle (Fig. 4a). TCA retrograde infusion increased adrenal MPO. 3-AB pretreatment significantly attenuated MPO increase in ANP rats (a trend at 3 h, and statistically significant reduction at 6–24 h; Fig. 4b).

TAC retrograde infusion caused severe destruction of sinusoids, hemorrhagic necrosis and various types of degeneration (vacuolar and granular degenerations, steatosis) in adrenal glands (Fig. 4e). 3-AB pretreatment apparently reduced histological damages (Fig. 4f and c).

### PARP inhibition reduced adrenocortical cell apoptosis and ultrastructure changes

**Fig. 5:**
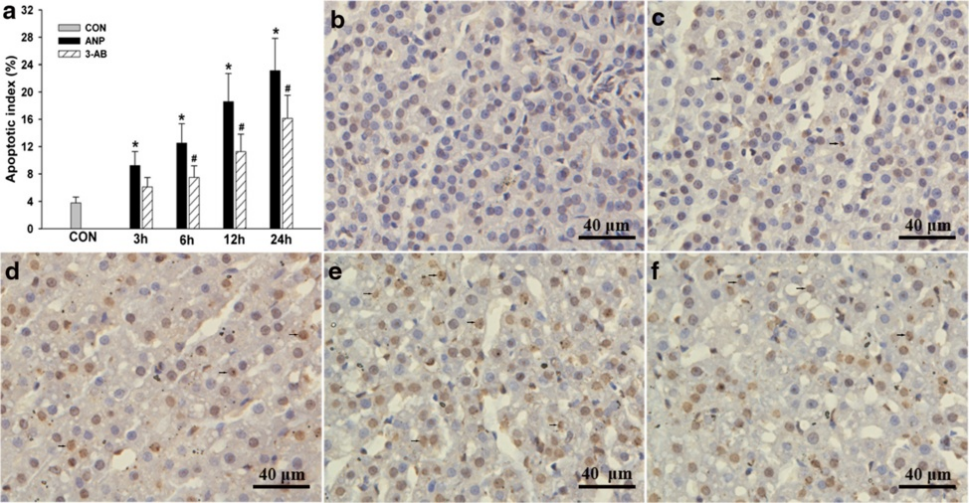
Representative figures of TUNEL-positive cells (*arrows*) after pancreatitis treated with vehicle or 3-AB. Nuclear staining of adrenocortical cells in control (**b**), ANP 3 h (**c**), ANP 6 h (**d**), ANP 12 h (**e**), 3-AB-treated 12 h (**f**) rats are shown. **a** Apoptotic index of adrenocortical cell. *P < 0.05 vs. CON group, ^#^ *P* < 0.05 vs. ANP group. Bar = 40 μm. (Original magnification × 400)

TUNEL assay revealed only few apoptotic cells in the adrenal glands from the sham control rats (Fig. 5). TAC retrograde infusion increased the number of apoptotic cells in adrenal cortex. 3-AB pretreatment significantly reduced the apoptosis index (Fig. 5a, f).

**Fig. 6:**
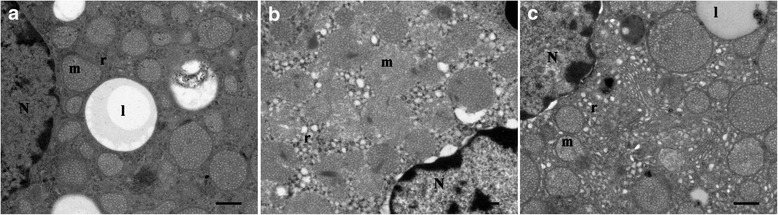
TEM of rat adrenocortical zona fasciculate cells at 12 h after pancreatitis. **a** Adrenocortical cells of CON group with normal nucleus (N) and mitochondria (m). Cytoplasm is filled with characteristic round mitochondria with tubulovesicular internal membranes, ample smooth endoplasmatic reticulums (r), and lipid droplets (l). **b** Adrenocortical cells of ANP rats in the zona fasciculata. The nucleus was seriously shrunk and dark-stained, chromatin was condensed, membrane of nucleus was tortuous. Some mitochondrion showed coagulation and mitochondrial crista disappeared or showed coagulation. Endoplasmic reticulum was dilated and swollen. **c** Treatment with 3-AB showed minimal irregularity in nucleus, mitochondria and endoplasmic reticulum. Bar = 6 μm. (TEM × 5000)

EM analysis demonstrated intact nuclei and uniform chromatin in the fascicular zone of adrenal cortex in sham control rats. Apoptosis was apparent at 12 h after TCA retrograde infusion. Characteristic features of apoptosis included: pyknotic nuclei, extreme chromatin condensation, morphology and nuclear membrane integrity loss, and cytoplasmic condensation. These ultrastructure alterations in adrenocortical cell were significantly attenuated by 3-AB treatment (Fig. 6a-c).

### PARP inhibition reduced adrenal PARP, NF-kB and cytokines activation

**Fig. 7:**
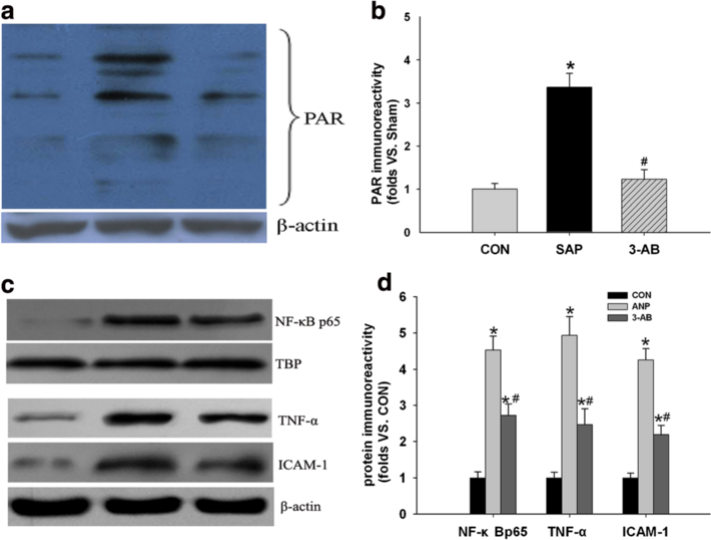
Western blot analysis of PAR, NF-kB, TNF-α and ICAM-1 of different groups. **a** The effect of inhibition on PAR after 12 h in pancreatitis animals pretreated with 3-AB. **b** Quantitative representation of the effects of 3-AB on PAR (*n* = 3). **c** The NF-kBp65 nuclear translocation, TNF-α and ICAM-1 activation increased in ANP group. After administration of 3-AB, the activation of NF-kB, TNF-α and ICAM-1 was significantly attenuated in adrenal. **d** Quantitative representation of the effects of 3-AB on the activation of NF-kB, TNF-α and ICAM-1. TBP (TATA-box binding protein) is a loading control of nuclear protein in Western blot analysis. *P < 0.05 vs. CON group, ^#^ *P* < 0.05 vs. ANP group

Rat model of acute adrenal injury at 12 h after ANP was selected as the successive study to determine the effect of 3-AB on PAR, because of progressive adrenal insufficiency and injury. The increase in PAR immunoreactivity caused by TCA retrograde infusion was attenuated by 3-AB (Fig. 7a-b). TCA retrograde infusion increased the expression of NF-kBp65, TNF-α and ICAM-1 in adrenal glands. 3-AB attenuated the activation of adrenal NF-kB, TNF-α and ICAM-1 in pancreatitis (Fig. 7c-d).

### PARP inhibition improved the survival rate of ANP rats

**Fig. 8:**
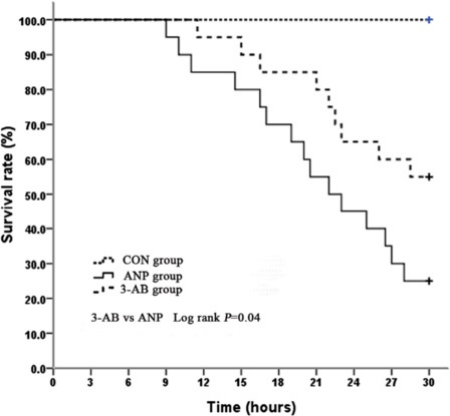
Effects of 3-AB on TCA-induced ANP mortality. Survival rate was estimated using the Kaplan-Meier method; different groups were compared using the log-rank test. *N* = 20 rats per group. ANP versus CON group, *P* = 0.00; 3-AB versus ANP group, *P* = 0.04; 3-AB versus CON group, *P* = 0.01

All animals in CON group survived the 30 h observation period. Nearly 80 % of these rats died within 30 h after ANP. 3-AB pretreatment decreased the mortality (Fig. 8).

## Discussion

The present study demonstrated that PARP inhibition with 3-AB could suppress inflammation and promotes recovery from adrenal injury in a rat model of ANP. The TCA retrograde infusion elevated serum corticosterone at the early stage. However, serum corticosterone then rapidly declined to a level below the baseline. Such a profile parallels adrenal insufficiency in many human patients with ANP. These findings suggest a critical role of PARP activation in the pathogenesis of acute adrenal injury.

Interestingly, PARP activation was time-dependent, with a prolonged time-course. Pathophysiological processes such as acute pancreatitis, generate free radicals and oxidant species lead to DNA injury and the activation of PARP [26, 27]. This delayed pattern of PARP activation may be related to the continuing presence of free radical and oxidant production in the injured adrenal (which would, therefore, maintain a continuing trigger for DNA single-strand breakage). Once an extensive amount of PAR polymer is formed, it remains significantly elevated for a prolonged period after a variety of toxic insults [28]. This would also explain the observation that the degree of pancreatitis-associated adrenal injury gradually increased with time.

Our study also demonstrated that the impairment of adrenal function and structure (indication of adrenocortical cell death) in ANP rats were significantly improved after PARP inhibition. Cell death appears to play a role in adrenocortical remodeling during embryonic and postnatal development of primates [29]. Recent publications suggest a role of PARP in necrosis. For example, necrosis caused by H_2_O_2_ in intestinal epithelial cells or fibroblasts is inhibited by PARP antagonists or by PARP-1 deficiency [30]. PARP-1 knockout mice are protected from endotoxin-induce lethality and hemorrhagic shock [31]. These data showed that protection from cell death was achieved by prevention of ATP depletion, which was caused by PARP activation. Apoptosis might represent a regulatory mechanism that allows adrenal gland to cope with stresses as well as functional demands [32]. In our study, apoptotic nuclei were observed in adrenal cortex of pancreatitis rats by TUNEL. In addition, our ultrastructural analysis of adrenocortical cells indicates that PARP inhibition attenuated adrenocortical cell apoptosis and improved ultrastructral changes. Steroidogenesis takes place in mitochondria and the smooth endoplasmic reticulum in the steroid-producing cells, depression of serum corticosterone was related to the direct adrenal injury which was characterized by a sequence of degeneration, hemorrhage, necrosis, and apoptosis in the adrenal cortex [1, 7, 33]. Neutrophils infiltration into inflamed tissue play a crucial role in the breakdown and remodeling of injured tissue [34]. We have demonstrated that rats respond to ANP with a significantly higher degree of tissue neutrophils infiltration in the adrenal gland. In our experiments, 3-AB significantly reduced adrenal MPO response to ANP protocol. It is important to note that in experimental AP, the glucocorticoid agonists attenuated the MPO activity. A recent publication underlines the importance of glucocorticoid action in the control of inflammatory mediator-induced remote organ damage during AP [35]. These results underlines that an appropriate adrenal glucocorticoid response to inflammatory stimuli is critical for the organism to cope with disease.

PARP activation contributes importantly to the pathophysiology of the inflammatory response. Soriano et al. reported significantly lower levels of TNF-α and IL-6 together with a reduced degree of cellular necrosis and organ inflammation in PARP-1 deficient mice with peritonitis [36]. A previous study in a secretagogue-induced murine model of acute pancreatitis [20] demonstrated that PARP-1 antagonism (or deficiency) could reduce damage to the pancreas and the lungs [26, 37]. Conceivably, the protective effects of PARP-1 antagonism in organs other than the pancreas could be secondary to alleviation of pancreatitis. However, we do believe the contribution of direct action of PARP-1. For example, suppression of PARP-1 expression by pharmacological inhibitor or the siRNA protects against oxidative stress-related injuries in cultured human hepatocyte WRL-68 cells [38].

Our findings indicated that PARP inhibition reduced the activation and the subsequent expression of pro-inflammatory genes (e.g., TNF-α and ICAM-1). PARP influences inflammation by interacting with the transcription factor NF-kB, in the absence of PAR formation. PARP inhibition protected animals from inflammatory injury, which is associated with the impaired transcription of many NF-kB-regulated pro-inflammatory factors [15]. Evidence suggests that these mediators can influence the synthesis of glucocorticoid by interfering the corticotropin-releasing hormone and adrenocorticotropic hormone activity [16]. Our study also demonstrates a significant impairment in adrenal glucocorticoid release in 12 h after pancreatitis. A prospective trial using low-dose glucocorticoid replacement for the treatment of sepsis showed a reduction in mortality with no increase in adverse events [39]. Increasing use of steroid replacement for treatment of sepsis in the intensive care unit has led critical care physicians to use steroids in other disease processes, such as trauma and hemorrhagic shock [3]. In severe pancreatitis, at least in the early phase, several animal experiments have shown improved survival after steroid treatment [2]. Last but not least, the mortality rate of rats in ANP was significantly reduced after PARP inhibition in our experiments, indicating clinical relevance. One limitation with this study is that, according to recent data, 3-AB is not a potent antioxidant [40]. However, we cannot exclude that some additional pharmacological effects of 3-AB, although unlike at used dosage, may have effect on observed results. The various classes of recently emerging potent, specific PARP inhibitors will help to further clarify this question.

## Conclusions

In conclusion, PARP overactivation may participate in a cascade of cellular and molecular events associated with of transcription factors activation in the adrenal after injury in ANP. Here, we demonstrate that PARP inhibition could protect against adrenal injury by blocking both adrenocortical cell necrosis/apoptosis and inflammatory factors, thereby collectively improves the impaired adrenal function. These findings encourage future studies of PARP as a site of intervention in the treatment of acute pancreatitis.

## Abbreviations

3-AB, 3-Aminobenzamide; ANP, acute necrotizing pancreatitis; ATP, adenosine triphosphate; NAD^+^, nicotinamide adenine dinucleotide; NF-kB, nuclear factor-kappaB; PAR, poly(ADP-ribosyl)ated protein; PARP, poly(ADP-ribose) polymerase; RAI, relative adrenal insufficiency; TCA, sodium taurocholate
